# BDNF signaling during the lifetime of dendritic spines

**DOI:** 10.1007/s00441-020-03226-5

**Published:** 2020-06-14

**Authors:** Marta Zagrebelsky, Charlotte Tacke, Martin Korte

**Affiliations:** 1 Division of Cellular Neurobiology, Zoological Institute, TU Braunschweig, Spielmannstr 7, 38106 Braunschweig, Germany; 2 Helmholtz Centre for Infection Research, AG NIND, Inhoffenstr. 7, D-38124 Braunschweig, Germany

**Keywords:** Brain-derived neurotrophic factor, Dendritic spines, Neurotrophin, TrkB, p75NTR

## Abstract

Dendritic spines are tiny membrane specialization forming the postsynaptic part of most excitatory synapses. They have been suggested to play a crucial role in regulating synaptic transmission during development and in adult learning processes. Changes in their number, size, and shape are correlated with processes of structural synaptic plasticity and learning and memory and also with neurodegenerative diseases, when spines are lost. Thus, their alterations can correlate with neuronal homeostasis, but also with dysfunction in several neurological disorders characterized by cognitive impairment. Therefore, it is important to understand how different stages in the life of a dendritic spine, including formation, maturation, and plasticity, are strictly regulated. In this context, brain-derived neurotrophic factor (BDNF), belonging to the NGF-neurotrophin family, is among the most intensively investigated molecule. This review would like to report the current knowledge regarding the role of BDNF in regulating dendritic spine number, structure, and plasticity concentrating especially on its signaling via its two often functionally antagonistic receptors, TrkB and p75^NTR^. In addition, we point out a series of open points in which, while the role of BDNF signaling is extremely likely conclusive, evidence is still missing.

## Introduction

Since their first description by Ramon y Cajal (yCajal 1891), dendritic spines have been postulated to be involved in regulating the communication between neurons. Indeed, these tiny membranous protrusions, emerging from the dendrites of most principal neurons, are the postsynaptic site of the majority of excitatory glutamatergic synapses in the brain (Gray 1959). Dendritic spines consist of a bulbous head, containing the postsynaptic density and connected to the dendrite via a thin and long neck. Dendritic spines serve as compartments in which calcium (Muller and Connor 1991) and biochemical (Guthrie et al. 1991) and electrical signals (Araya et al. 2006; Grunditz et al. 2008) are confined during glutamatergic transmission, shaping synaptic transmission.

Dendritic spines come in diverse sizes and morphologies especially regarding head volume, spine neck lengths, and thickness and are commonly classified, according to these criteria in three groups, i.e., stubby, thin, and mushroom spines (Peters and Kaiserman-Abramof 1969), possibly reflecting different functions. The size of the spine head scales with the size of the postsynaptic density, the number of neurotransmitter receptors, and synaptic strength. Moreover, the observation that dendritic spines are extremely dynamic both in size and shape of pre-existing spines and also in their new formation and disappearance links them to processes of activity-dependent synaptic plasticity (Sala and Segal 2014). Indeed, several studies showed activity-dependent changes in dendritic spine morphology and number occurring after induction of synaptic plasticity, i.e., long-term potentiation (LTP) and long-term depression (LTD). Our current knowledge about dendritic spines indicates their crucial role in synaptic transmission and plasticity linking their morpho-physiology with cognition processes such as acquisition of new information and its long-term retention (Holtmaat and Caroni 2016). Accordingly, spine dysfunction is related to cognitive decline in aging (von Bohlen und Halbach et al. 2006) as well as to several neuropsychiatric, neurodevelopmental, and neurodegenerative diseases including autisms (Sudhof 2008), mental retardation (Purpura 1974), and Alzheimer’s disease (Dorostkar et al. 2015). Ensuring their crucial physiological functions and preventing the severe consequences of their dysfunction under pathological conditions require a very tight regulation of the processes involved in the different phases of the life of dendritic spines. Among the many molecules controlling the structure and function of dendritic spines, the brain-derived neurotrophic factor (BDNF) stands out for its compelling activities at all stages of a spine’s life.

This review will give an overview about the actions of BDNF signaling, via its receptors in regulating the dendritic spine formation, maturation, and its need for the maintenance of the mature spine phenotype and their plasticity. BDNF is a member of the neurotrophin family, comprising four closely related secreted proteins known to regulate survival, growth, and differentiation of neurons during development as well as activity-dependent synaptic plasticity and processes of learning and memory in the mature CNS (Park and Poo 2013; Zagrebelsky and Korte 2014). Both BDNF and its precursor proBDNF have been shown to be biologically active and exert their actions upon their binding to two transmembrane receptors – the tropomyosin receptor tyrosine kinase B (TrkB), with a higher affinity for the mature form of BDNF, and the p75 Neurotrophin receptor (p75^NTR^) preferentially binding proBDNF (Barbacid 1993; Chao and Hempstead 1995).

BDNF is one of the neuroprotective, growth substances released by neurons under stress or pathological conditions, and brain pathologies are associated with the reduction in BDNF release, resulting in lower brain ad blood levels. Thus, BDNF has been suggested as a biomarker for different brain pathologies and for the efficacy of their therapy. Indeed, most currently used treatments are accompanied by significant changes in BDNF expression and release levels. However, due to space limitations, this review will not address the current knowledge regarding BDNF signaling in the pathophysiology and therapy of neurological diseases.

## The role of BDNF in regulating dendritic spine development

### Spinogenesis

**Fig. 1:**
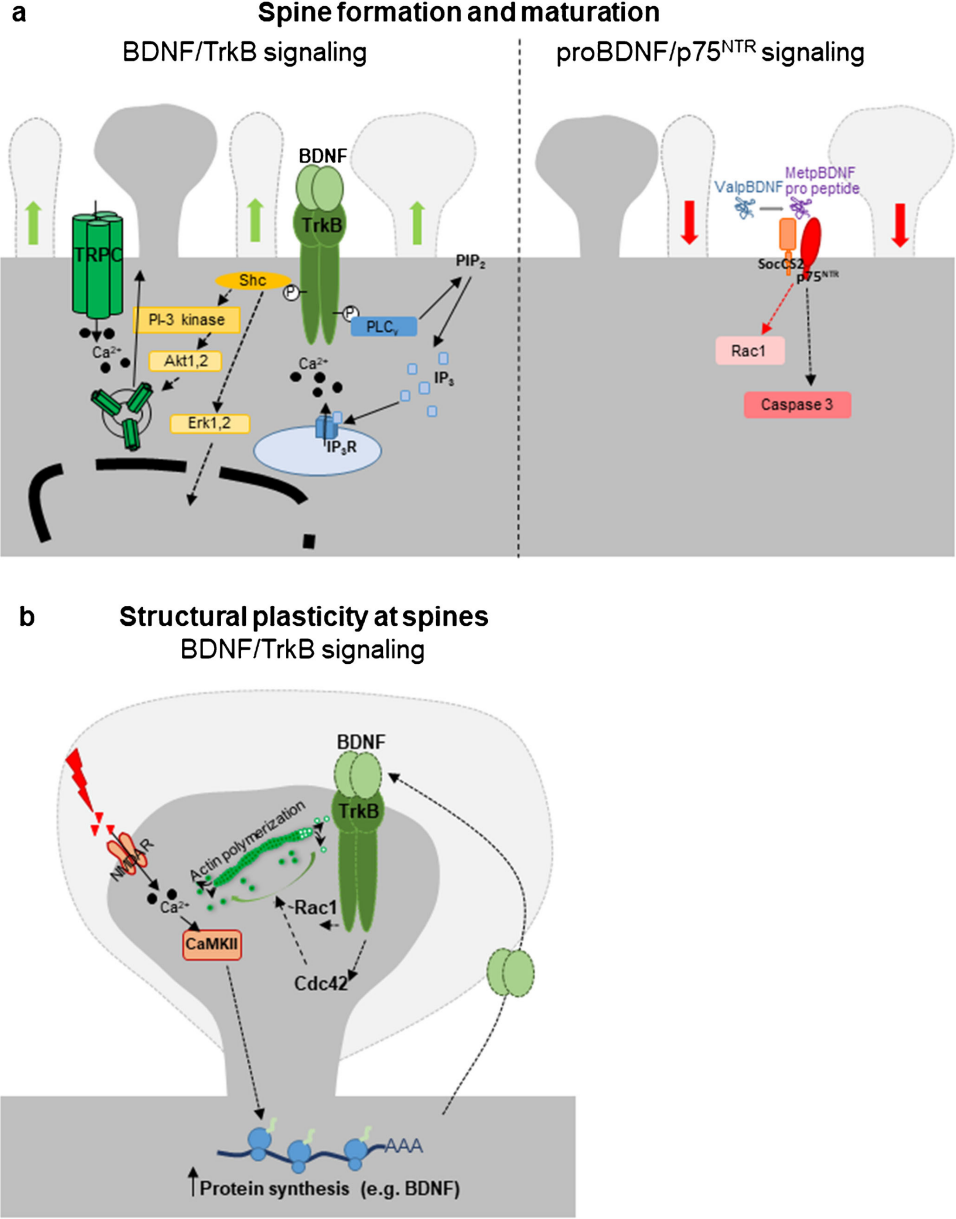
**a** Schematic representation of the intracellular signaling cascades downstream of the binding of BDNF to TrkB and of its precursor proBDNF to p75^NTR^. BDNF binding to TrkB promotes dendritic spine formation and maturation via the Shc site Erk1/2, to control gene regulation as well as the activation of the PI-3 kinase in order to promote the insertion of TRPC channels at the membrane. Moreover, activation of the PLCγ site promotes the formations of IP_3_ and the release of Ca^2+^ from the internal stores. On the contrary, binding of Met variant of proBDNF or of the BDNF pro-peptide results in dendritic spine loss via the inhibition of Rac1 and the activation of caspase-3. **b** Schematic representation of the signaling initiated by BDNF binding to TrkB and resulting in the long-lasting enlargement of the spine head in a process known as structural potentiation (sLTP). BDNF binding to TrkB induces in this case the polymerization of actin by promoting the activity of Rac1 and Cdc42 within dendritic spines. Moreover, activation of the NMDARs results in an increase in local protein synthesis in a CaMKII-dependent manner possibly also providing the BDNF required to activate TrkB at spines in an autocrine way. Black arrows indicate activation, while the red arrows indicate inhibition

Mature dendritic spines have been proposed to develop from filopodia, long thin and highly motile dendritic protrusions upon their contact with an axon (Bonhoeffer and Yuste 2002). The maturation process of dendritic spines involves the progressive increase in their density, associated to a reduction in the number of filopodia (Dunaevsky et al. 1999; Nimchinsky et al. 2002). Several studies have shown that long-term in vitro treatment with exogenous BDNF increases dendritic spine density in pyramidal hippocampal neurons (Gottmann et al. 2009; Ji et al. 2005; Tyler and Pozzo-Miller 2001; for reviews see Zagrebelsky and Korte 2014) and in cerebellar Purkinje cells (Shimada et al. 1998). These growth-promoting effects of BDNF occur in a TrkB-dependent manner. Indeed, activation of TrkB is induced by BDNF application protocols known to increase dendritic spine density (Ji et al. 2010), and the increase in spine density upon BDNF treatment is prevented by simultaneously inhibiting the Trk receptors using K252a (Tyler and Pozzo-Miller 2001) or more specifically by TrkB receptor bodies (Shimada et al. 1998). Accordingly, activation of signaling pathways downstream of TrkB has been shown to be involved in the effects of BDNF on spine formation (Fig. 1a). The BDNF-induced increase in spine density depends upon the membrane insertion of transient receptor potential canonical subfamily 3 (TRPC3) channels promoted by the activation of the TrkB-PLCγ pathway (Amaral and Pozzo-Miller 2007). Also, the activation of MAPK/ERK1/2, downstream of the TrkB-Shc site, is required for the increase in dendritic spine density upon BDNF treatment in hippocampal pyramidal neurons (Alonso et al. 2004). Here it should be pointed out that the effects of an exogenous BDNF application on spine density are not reproduced in all studies, possibly due to different culture conditions (Kellner et al. 2014; Zagrebelsky et al. 2018). Indeed, culture conditions have been shown to influence the neuronal response to BDNF (Chapleau et al. 2008).Nevertheless, the physiological relevance of the positive effects of BDNF/TrkB signaling on dendritic spine formation in vitro is supported by the observation that scavenging endogenous BDNF in primary hippocampal cultures results in a decrease in spine density (Kellner et al. 2014). Moreover, inhibition of TrkB receptor activation with K252a not only blocked the effect of BDNF but further reduced spine density below control level (Tyler and Pozzo-Miller 2001).

Studying the role of BDNF/TrkB signaling in regulating dendritic spine formation in vivo is complicated by the fact that most of the *bdnf* knockout mice die soon after birth (Jones et al. 1994). Moreover, the conditional deletion of *bdnf* resulted in contradictory observations. On one side, a forebrain-specific *bdnf* knockout mouse (CamKII-BDNF^KO^), in which BDNF is lost primarily in the cortex and hippocampus during early adulthood showed a delayed decrease in dendritic spine density for layer II/III pyramidal neurons of the visual cortex (Vigers et al. 2012) suggesting a role of BDNF in maintaining dendritic spines rather than in their formation. On the other hand, the complete brain-specific deletion of *bdnf* in the Tau-BDNF^KO^ mouse showed a significant reduction in spine density for medium spiny neurons (MSNs) of the striatum, but not effects on the spines of hippocampal pyramidal neurons (Rauskolb et al. 2010). Interestingly, the potassium-chloride cotransporter KCC2 has been recently shown to increase dendritic spine density in a BDNF-dependent manner in the cortex but not in the hippocampus (Awad et al. 2018) further underlying the possibility that in vivo the BDNF actions on dendritic spine formation occur in an area-specific manner.

Most of the studies addressing the role of BDNF signaling in regulating dendritic spine formation have been performed in the hippocampus or the cortex. However, more recently the levels of both BDNF and TrkB have been shown to be regulated in the nucleus accumbens shell (NACsh) in a mouse model for chronic cocaine addiction (Graham et al. 2007; Graham et al. 2009). Moreover, loss-of-function for either BDNF or TrkB specifically in the NACsh results in reduction in cocaine self-administration (Graham et al. 2007; Graham et al. 2009) suggesting an important role of BDNF/TrkB signaling in this context. Psychostimulant drugs induce a series of biochemical and morphological alterations especially in the brain monoamine systems. Treatment with amphetamine or cocaine increases dendritic spine density of MSNs in NACsh and on apical dendrites of layer V pyramidal cells in the prefrontal cortex (Robinson and Kolb 1999). BDNF signaling through TrkB in NACsh is necessary for cocaine-induced long-lasting dendritic spine formation in MSNs (Anderson et al. 2017). Intriguingly, while increasing TrkB expression after chronic cocaine administration reverses the increase in dendritic spine density, loss-of-function for TrkB after chronic cocaine self-administration failed to affect spine density (Anderson et al. 2017) indicating an important role of BDNF/TrkB signaling in the cocaine-induced formation of new spines, but not in their maintenance.

### Dendritic spine maturation and stabilization

Dendritic spine maturation is characterized morphologically by an overall decrease in spine length and motility (Dailey and Smith 1996; Marrs et al. 2001) and an increase in spine head volume correlated to an enlargement of the postsynaptic density and to an increase in the number of inserted neurotransmitter receptors and scaffold proteins (Harris et al. 1992; Harris and Stevens 1989). These changes are reflected in the proportion of the different spine types, classified based on their morphology by confocal imaging. While thin spines, with their long necks and thin head, are supposed to represent a more immature stage, stubby and mushroom spines are characterized by larger postsynaptic densities and higher stability and therefore proposed to be mature spines (Bonhoeffer and Yuste 2002). Loss- and gain-of-function experiments for BDNF in vitro affect dendritic spine morphology, thereby altering the distribution of different spine types. Specifically, BDNF treatment in organotypic hippocampal cultures resulted in an increase in the proportion of stubby and a reduction in the one of mushroom spines (Tyler and Pozzo-Miller 2003). Moreover, acute and gradual BDNF application in primary hippocampal neurons resulted respectively in a fast and transient activation of TrkB and ERK1/2 associated to spine head enlargement or in a sustained TrkB and ERK1/2 activation accompanied by spine neck elongation (Fig. 1a; Ji et al. 2010). These observations underlie the importance of how BDNF itself is delivered. Moreover, it is important to point out that the effects of an exogenous BDNF application on dendritic spine maturation in hippocampal neurons depend on neuronal activity. When BDNF was applied together with botulinum toxin C to block miniature synaptic neurotransmitter release, the proportion of long and thin possibly immature spines was increased (Tyler and Pozzo-Miller 2003). Also, application of BDNF to hippocampal cultures kept in a high magnesium containing medium showed a significant increase in the proportion of mature spines with larger heads with a comparable significant decrease in the proportion of immature spines with small heads. Accordingly, a loss-of-function for BDNF by application of BDNF scavenging antibodies to primary hippocampal neurons resulted in a decrease in dendritic spine head width associated to an increase in length, indicating a less mature phenotype (Kellner et al. 2014). In addition, primary hippocampal and cortical pyramidal neurons show a significant decrease in the proportion of mushroom spines, associated to an increase in one of the thin spines upon cre-mediated deletion of *bdnf* both in vitro (Zagrebelsky et al. 2018) and in vivo (Rauskolb et al. 2010). In vivo, hippocampal pyramidal neurons of a mutant mouse specifically lacking the dendritic localization of BDNF (*bdnf* ^klox/klox^) show a higher density of longer and thinner dendritic spines in layer 2/3 pyramidal neurons of the visual cortex and in the hippocampus (An et al. 2008). Furthermore, knocking down specifically the long 3′ UTR, dendritic *Bdnf* mRNA or blocking its transport to dendrites inhibits spine maturation and pruning, whereas overexpressing it enhances these processes in cultured hippocampal neurons (Orefice et al. 2013).

Several lines of evidence indicate that the role of BDNF in promoting dendritic spine maturation depends on its signaling via TrkB. Expression of a dominant negative TrkB receptor in pyramidal neurons of the visual cortex resulted in the reduced maintenance of mushroom spines accompanied by an increase in the density of long and thin spines (Chakravarthy et al. 2006). Also, a recent study showed that copine-6, a C2-domain-contaning protein mediating calcium-dependent binding to membrane phospholipids, is recruited to spines upon BDFN application and promotes BDNF-dependent changes in dendritic spine morphology by recycling activated TrkB receptors to the membrane surface (Burk et al. 2018).

Overexpression of the postsynaptic density scaffold protein PSD-95 drives the maturation of glutamatergic excitatory synapses and increases dendritic spine density (El-Husseini et al. 2000). In cortical pyramidal neurons, the localization of PSD-95 at spines correlates with an increase in their stability over time (Cane et al. 2014). Interestingly, the TrkB receptor has been shown to be associated to PSD-95, and its activation, upon BDNF binding, increases the recruitment of the postsynaptic density protein to synapses via the PI3K pathway (Yoshii and Constantine-Paton 2007). Furthermore, BDNF/TrkB signaling increases PSD-95 localization at dendritic spines by prolonging the microtubule invasions (Hu et al. 2011). PSD-95 binds many postsynaptic molecules that can regulate dendritic spine growth. For example, PSD-95 binds Kalirin, a neuronal Rho guanine nucleotide exchange factor (Rho-GEF) that facilitates actin polymerization within spines, thereby increasing both their number and their size (Penzes et al. 2001). Taken together these observations suggest a crucial role of BDNF signaling via its TrkB receptor in promoting dendritic spine maturation and stabilization. On the other hand, it has to be mentioned that in developing pyramidal neurons in organotypic slice cultures of ferret visual cortex, the overexpression of BDNF resulted in an increase in the remodeling of dendritic spines, possibly acting through an autocrine loop (Horch et al. 1999).

### Role of BDNF signaling in activity-dependent structural plasticity at spines

Long-term synaptic plasticity, i.e., LTP and LTD, represents the cellular mechanism underlying learning and memory processes. Both LTP and LTD have been shown to be associated to structural changes at dendritic spines (Engert and Bonhoeffer 1999; Yuste and Bonhoeffer 2001). Specifically, LTP induction results in long-lasting spine head enlargement (Matsuzaki et al. 2004), while LTD results in its shrinkage (Nagerl et al. 2004; Zhou et al. 2004; for a review see Holtmaat and Svoboda 2009) reflecting changes in the number of neurotransmitter receptors (Kopec et al. 2006) and in the spine responsiveness to glutamate (Matsuzaki et al. 2004). While in vivo dendritic spines have been shown to remain stable over months (Grutzendler et al. 2002; Trachtenberg et al. 2002), providing the structural correlate for the long-term storage of information, structural plasticity of dendritic spines is required for learning and memory formation (Hayashi-Takagi et al. 2015; Xu et al. 2009; Yang et al. 2014a; Yang et al. 2009a). Structural plasticity at spines relies upon the activation of a series of intracellular signaling cascades mostly regulating the remodeling of the actin cytoskeleton and protein synthesis. Among the extracellular factors impinging on these intracellular signaling cascades, BDNF is for several reasons of special interest. BDNF is released by neurons in an activity-dependent manner (Goodman et al. 1996; Griesbeck et al. 1999) and specifically upon LTP-inducing electrical stimulation in hippocampal neurons (Gartner and Staiger 2002). Moreover, BDNF signaling modulates dendritic spine morphology and is required both for induction and maintenance of LTP (Korte et al. 1995; Kovalchuk et al. 2002; Zagrebelsky and Korte 2014) and for learning and memory processes (Alonso et al. 2002; Petzold et al. 2015). Recent evidence implicates BDNF in regulating activity-dependent structural plasticity at spines as BDNF/TrkB signaling is necessary and sufficient to induce long-lasting structural changes at dendritic spines upon LTP induction (Tanaka et al. 2008). Synaptic stimulation by glutamate uncaging was paired with postsynaptic spikes, a protocol resulting in the gradual and long-lasting spine head enlargement also known as structural LTP (sLTP; Matsuzaki et al. 2004). Here the progressive and long-lasting spine head enlargement upon pre- to postsynaptic pairing was shown to depend on TrkB (Tanaka et al. 2008). While the secretion of BDNF could not be directly shown, the authors suggested an autocrine function of BDNF released from the postsynaptic neuron in this context (Fig. 1b). More recently, fluorescence resonance energy transfer-based sensor for TrkB and two-photon fluorescence lifetime imaging were combined to monitor TrkB activity at single dendritic spines of CA1 pyramidal neurons upon sLTP. The results obtained describe in stimulated spines a fast, followed by a sustained activation of TrkB depending on the postsynaptic synthesis of BDNF and its release at single spines (Harward et al. 2016). While these results come with the limitation that they are based on the ectopic overexpression of pHluorin-fused BDNF and possibly do not represent the endogenous conditions, they are noteworthy in supporting a crucial role of postsynaptic BDNF/TrkB signaling not only for functional but also for structural plasticity at spines. Indeed, BDNF has been shown to be a newly a synthetized product required for the maintenance of late LTP (Pang et al. 2004).

A dense network of actin cytoskeleton underlies the structure of dendritic spines and via its dynamics supports the structural changes at spines during plasticity processes (Colgan and Yasuda 2014; Hotulainen and Hoogenraad 2010). Cytoskeletal remodeling depends upon the activation of the small GTPase proteins (Nakahata and Yasuda 2018). Specifically, the precise spatiotemporally coordinated activation of RhoA, Rac1, and Cdc42 downstream of the Ca^2+^/calmodulin kinase II (CaMKII; Lee et al. 2009; Matsuzaki et al. 2004) underlies sLTP (Bosch et al. 2014; Murakoshi et al. 2011). BDNF signaling modulates the activation of actin binding proteins (Fass et al. 2004; Gehler et al. 2004) downstream of Rho GTPases (Briz et al. 2015). Indeed, exogenous application of BDNF signaling via TrkB in rat hippocampal slices promotes actin polymerization resulting in an increase in the number of dendritic spines containing F-actin, and co-application of TrkB receptor bodies prevents the actin polymerization induced by LTP-inducing theta burst stimulation (Rex et al. 2007). These results indicate that BDNF is a crucial component in promoting LTP-related cytoskeletal changes at dendritic spines. A recent study confirmed this hypothesis by showing the critical role of BDNF in inducing the coordinated activation of Rho GTPases during sLTP (Hedrick et al. 2016). Specifically, BDNF signaling via TrkB postsynaptically activates Cdc42 and Rac1, but not RhoA (Hedrick et al. 2016). Interestingly, this action of BDNF supports the input specificity and the heterosynaptic facilitation typically observed in sLTP by activating on one side a spine-specific signaling comprising BDNF–TrkB–Cdc42, and a diffuse one comprising BDNF–TrkB–Rac1 signaling. Moreover, these results enlighten the facilitation of plasticity observed upon BDNF treatment by priming spines to sLTP via the activation of Rho GTPases.

Sustained structural plasticity of spines requires activity-dependent protein synthesis (Bosch et al. 2014; Govindarajan et al. 2011; Tanaka et al. 2008). For several plasticity-relevant proteins (i.e., β-actin and CaMKII; Miller et al. 2002; Tiruchinapalli et al. 2003), the mRNAs are bidirectionally transported along microtubule in dendrites in an activity-dependent manner and stop at the base of stimulated spines suggesting their regulated local translation upon sLTP induction (Buxbaum et al. 2015). While it is not yet conclusively shown that BDNF promotes dendritic spine morphogenesis upon activity-dependent plasticity by regulating local protein synthesis, different lines of evidence suggest this possibility. BDNF is now clearly implicated in promoting local protein synthesis by the observations that its application increases the incorporation of radio labeled amino acids and promotes the translation of a fluorescent translation reporter also in isolated dendrites and in synaptosomes (Aakalu et al. 2001; Takei et al. 2004). Moreover, BDNF application induces a protein synthesis-dependent form of LTP (Kang and Schuman 1996; Kang and Schuman 1995) opening the possibility that BDNF promotes structural plasticity at spines both by promoting the cytoskeletal reorganization and the local protein synthesis of plasticity promoting proteins.

### Involvement of BDNF signaling in learning-induced structural plasticity in vivo

A role of BDNF in regulating learning and memory processes has been suggested by several studies often based on purely correlative evidence. Indeed, BDNF and TrkB expression (Gomez-Pinilla et al. 2001; Hall et al. 2000; Silhol et al. 2007) and TrkB phosphorylation (Gooney et al. 2002) are rapidly induced in the hippocampus upon contextual spatial learning. And, fear extinction training has been shown to increase BDNF expression in the ventral hippocampus (Peters et al. 2010; Rosas-Vidal et al. 2014). Gain-of-function approaches for BDNF and TrkB in the hippocampus improve spatial learning performance (Cirulli et al. 2004; Koponen et al. 2004; Nakajo et al. 2008) and in the infralimbic cortex facilitate fear extinction (Peters et al. 2010). On the contrary, interfering with the BDNF/TrkB signaling results in impaired performance in the water maze task (Mu et al. 1999; Petzold et al. 2015) and prevents fear extinction (Peters et al. 2010; Rosas-Vidal et al. 2014) or consolidation (Chhatwal et al. 2006). Learning and memory processes are associated with structural alterations at dendritic spines in the hippocampus, cortex, and amygdala (Heinrichs et al. 2013; Lai et al. 2012; Leuner and Shors 2004; Moser et al. 1994; Vetere et al. 2011a; Vetere et al. 2011b), and BDNF modulates dendritic spine number and structure both during development and in the adult brain. However, while the evidence for a role for BDNF in promoting structural plasticity at spines in vivo during learning and memory processes is still largely correlative, one recent study provided direct evidence for a role of BDNF signaling in promoting formation of new dendritic spines upon motor learning. This study analyzed the role of microglia produced BDNF in this context. Although the major source of BDNF in the adult brain appears to be neurons (Rauskolb et al. 2010; Dieni et al. 2012), BDNF can also be detected in oligodendrocytes, astrocytes, and microglia (Dougherty et al. 2000). Conditional deletion of BDNF from microglia resulted in the impairment of motor learning associated to an impairment in the learning-induced formation of new dendritic spines in the motor cortex (Parkhurst et al. 2013) suggesting a new BDNF-mediated role for microglia in locally modulating specific subsets of synaptic connections possibly involved in specific learning tasks.

### Involvement of BDNF in age-dependent dendritic spine alterations

Aging in the CNS is a physiological process characterized by the progressive decrease in brain volume and decline in brain function leading to different degrees of cognitive impairment. The cognitive decline seems not to reflect neuronal loss, occurring in fact only in few brain areas (Cabello et al. 2002; Stranahan et al. 2012; Woodruff-Pak et al. 2010), but rather subtle structural changes in network connectivity at the level of dendritic spines. An age-related progressive loss of dendritic spines was shown for the cerebral cortex and the hippocampus of rodents, non-human primates, and humans (Dumitriu et al. 2010; Feldman and Dowd 1975; Jacobs et al. 1997; Luebke et al. 2010; von Bohlen und Halbach et al. 2006) and is correlated to age-related cognitive impairment in Rhesus monkeys (Dickstein et al. 2013) and impairment of hippocampus-dependent spatial learning in aged rodents (von Bohlen und Halbach et al. 2006; Zeng et al. 2012b). Moreover, cognitively unimpaired aged rats show no alterations in spine density (Zeng et al. 2012b) strengthening the functional relevance of the age-dependent spine loss. Moreover, some studies described alterations in the proportion of spine types with a predominance of larger spines in older animals (Xu et al. 2018) and an increase in their stability (Mostany et al. 2013). This observation is relevant to the cognitive impairment as smaller, thin spines have been shown to be especially important for memory storage (Bourne and Harris 2007).

Impairments in the BDNF/TrkB signaling are highly correlated with cognitive impairment and dendritic spine changes during aging. Indeed, TrkB expression and activation have been shown to be reduced in aged rodents and humans (Buhusi et al. 2017; Croll et al. 1998; Gooney et al. 2004; Webster et al. 2006). However, there is less consensus about the effect of aging on BDNF levels. In humans, although increasing age in health individuals has been associated with reduced levels of serum BDNF and poorer memory performance (Shimada et al. 2014; Siuda et al. 2017), no changes in BDNF mRNA in the hippocampus and temporal cortex were detected from postmortem brains (Webster et al. 2006). While in mice, BDNF levels did not change in the hippocampus with age (Buhusi et al. 2017), aged cognitively impaired rats displayed a lower increase in training-induced BDNF mRNA level than aged non-impaired rats in the CA1 region of the hippocampus (Schaaf et al. 2001). Furthermore, BDNF-induced LTP and its downstream signaling were significantly impaired in aged rats (Gooney et al. 2004). Accordingly, aged mutant mice carrying a deletion in one copy of the BDNF gene performed significantly worse than controls (Linnarsson et al. 1997). Cognitive unimpaired aged rats show no alterations in spine density associated to normal levels of TrkB expression, activation, and downstream signaling (Zeng et al. 2012b), and lower levels of TrkB expression, activation, and downstream signaling are associated with lower spine density and impaired hippocampus-dependent learning in aged rats (Zeng et al. 2012b). Intriguingly, the cognitive impairment as well as the decrease in spine number could be rescued by a treatment with the TrkB agonist 7,8-sihydroxyflavone (7,8-DHF; Zeng et al. 2012b) strengthening the link between BDNF/TrkB signaling and spine loss upon aging. Moreover, proBDNF and P75^NTR^, exerting a negative regulation on dendritic spine density and plasticity, are upregulated in aged mice (Buhusi et al. 2017; Costantini et al. 2005; Perovic et al. 2013), and hippocampal proBDNF levels are inversely correlated with spatial memory in aged mice (Buhusi et al. 2017). In spite of the correlative evidence for a role of the age-dependent alteration in BDNF/TrkB signaling and dendritic spine loss, studies causally linking these two events are still lacking, which would be a prerequisite for a rational therapeutic intervention using BDNF or TrkB agonists.

### Autocrine versus local paracrine BDNF actions

BDNF is a sticky, positively charged protein whose biochemical characteristics prevent its diffusion within the target region and indicate that BDNF is only acting locally at synapses (few micrometers range; Horch and Katz 2002). Moreover, defining the locus of BDNF functional secretion is made by difficult its very low endogenous amounts. Taken together the low endogenous amounts and the locally restricted actions of BDNF complicate the distinction between autocrine and local paracrine signaling. However, a few studies made use of chimera neuronal cultures to address this issue in the context of the activity of BDNF on neuronal architecture and dendritic spines and provided strong evidence for a cell autonomous, autocrine mode of action for BDNF in this context. Particularly, a sparse deletion of BDNF in adult born granule cells of the dentate gyrus resulted in shorter dendrites and impaired spino- and synaptogenesis in a cell autonomous manner (Wang et al., 2015). Accordingly, BDNF overexpression in pyramidal neurons of the visual cortex induced structural instability specifically of dendrites and spines in BDNF expressing neurons (Horch et al. 1999). On the other hand, the same authors also showed locally restricted increase in dendrite density upon BDNF overexpression in neighboring, non-transfected neurons, depending on BDNF release indicating a local paracrine action of BDNF (Horch and Katz 2002). These observations suggest the possibility of a self-amplifying autocrine BDNF signaling as shown at early stages of axonal development (Cheng et al. 2011). Finally, recent studies convincingly demonstrated a spine-autonomous, autocrine mode of action for endogenous BDNF in regulating structural LTP at a single spine level (Harward et al., 2016; Hedrick et al. 2016) as well as theta burst-induced LTP (Edelmann et al., 2015; Brigadski and Lessmann 2020).

### BDNF/TrkB and proBDNF/p75^NTR^: functional antagonisms in regulating dendritic spine structure and plasticity

BDNF is initially synthetized as a precursor proBDNF which is cleaved to produce the mature protein exerting a series of positive actions on neuronal structure and plasticity processes via its specific binding to TrkB. While p75^NTR^ also binds the mature BDNF, albeit at a lower affinity, it preferentially mediates the action of proBDNF (Lee et al. 2001). While the BDNF/TrkB-induced modulation of dendritic spine structure and plasticity is well described (see above; Minichiello 2009; Zagrebelsky and Korte 2014), the role of proBDNF/p75^NTR^ signaling in this context remains less investigated. The application of exogenous uncleavable proBDNF (CR-proBDNF) in vitro suggests that it exerts opposite effects than mature BDNF (Cowansage et al. 2010; Lu et al. 2005; Teng et al. 2010). While the treatment of mature hippocampal neurons with BDNF increases their dendritic spine density, application of CR-proBDNF significantly reduced it without affecting neuronal survival (Koshimizu et al. 2009). These opposite actions of proBDNF and BDNF on dendritic spines are complemented by the observation that the actions of exogenous BDNF on the pyramidal neurons of slice cultures depend on the presence of serum in the medium. Indeed, while BDNF exposure of slices kept in serum-free conditions increases the proportion of stubby spines, in serum-containing media, the same treatment induces an increase in the proportion of mushroom and thin spines and a decrease of one of the stubby spines (Chapleau et al. 2008). Intriguingly, slices maintained in serum media showed a lower p75^NTR^-to-TrkB expression level than serum-free slices (Chapleau et al. 2008) supporting the idea of the opposing functional signaling by on the one hand of proBDNF/p75^NTR^ and on the other hand of BDNF/TrkB ligand-receptor interaction. Accordingly, pyramidal neurons of the hippocampus of p75^NTR^ knockout mice have a significantly higher dendritic spine density associated with a decrease in the proportion of stubby spines (Zagrebelsky et al. 2005). However, whether proBDNF is secreted by neurons in vivo under physiological conditions, it is still under debate. While some studies showed that BDNF and its pro-peptide are stored in presynaptic dense core vesicles and are secreted together (Dieni et al. 2012) suggesting an intracellular cleavage (Matsumoto et al. 2008), others showed proBDNF release by neurons (Yang et al. 2009b) and its activity-dependent extracellular cleavage (Nagappan et al. 2009; Pang et al. 2004). It is noteworthy that the physiological relevance of the proBDNF/p75^NTR^ signaling in neuronal plasticity is supported by the observation that a low-frequency stimulation results in the release of proBDNF (Nagappan et al. 2009) leading to the p75^NTR^-dependent facilitation of LTD (Woo et al. 2005). Moreover, proBDNF/p75^NTR^ signaling was shown to mediate the synaptic depression observed in neighboring, non-coactive spines upon strengthening of synaptic connections via spontaneous activity in the hippocampus (Winnubst et al. 2015).

To further evaluate the role of the endogenous proBDNF in vivo on dendritic spines in depth, a knockin mouse was generated expressing one mutated, uncleavable *probdnf-HA* allele (*probdnf-HA*/+; Yang et al. 2014b) resulting in the specific secretion of proBDNF upon neuronal stimulation. In *probdnf-HA*/+ mice, a significant decrease in dendritic spine density was observed which was greater than the one shown by heterozygous *bdnf* knockout mice indicating that proBDNF exerts specific negative effects on dendritic spines density (Yang et al. 2014b). Moreover, while in hippocampal neurons the constitutive somatic synthesis of BDNF promotes dendritic spine formation in a TrkB-dependent manner (An et al. 2008; Orefice et al. 2013), neuronal activity promotes the translation of dendritic *bdnf* mRNA and the secretion of its translation product mostly as proBDNF (Orefice et al. 2016). The proBDNF secreted under these conditions binds to p75^NTR^ resulting in increased dendritic spine pruning (Orefice et al. 2016). While no data so far show a role of proBDNF in modulating structural plasticity at spines in *probdnf-HA*/+ mice, theta burst-induced LTP is impaired and LTD is enhanced (Yang et al. 2014b) showing its ability to modulate synaptic plasticity.

It was generally believed that after cleavage, the BDNF pro-peptide is rapidly degraded. But it was shown that in the hippocampus, the mature BDNF, and its pro-peptide are stored together in dense core vesicles and are secreted in equimolar ratios at a ten times higher concentration than proBDNF (Dieni et al. 2012). The secretion of the BDNF pro-peptide from hippocampal neurons in vitro occurs in an activity-dependent manner (Anastasia et al. 2013; Guo et al. 2016; Mizui et al. 2015). A common single-nucleotide substitution in the human *bdnf* gene results in a Val66Met substitution in the BDNF pro-peptide sequence and has been shown to affect activity-dependent BDNF secretion and to be associated with a decrease in hippocampal volume, impairment of episodic memory, and increase in depression and anxiety disorders (Chen et al. 2006; Egan et al. 2003; Soliman et al. 2010; Verhagen et al. 2010). Recently the Met66 variant of the BDNF pro-peptide has been identified as a new, biologically active ligand able to modulate neuronal morphology and plasticity. Application of Met66 BDNF pro-peptide to hippocampal neurons resulted in growth cone collapse in a p75^NTR^-dependent manner via its interaction with the sortilin-related Vps10p-domain sorting receptor 2 (SorCS2) known to facilitate the interaction of p75^NTR^ with downstream signaling proteins (Anastasia et al. 2013). Moreover, exposure of mature hippocampal neurons to BDNF pro-peptide significantly reduces dendritic spine density via the activation of caspase-3 (Guo et al. 2016). And treatment with purified recombinant BDNF pro-peptide significantly enhances LTD in a p75^NTR^-dependent manner by promoting NMDS-triggered GluA2 endocytosis (Mizui et al. 2015). Interestingly, while the Val66 BDNF pro-peptide enhanced LTD, the Met66 variant markedly reduced it (Mizui et al. 2015). Together with the observation that the Met66 BDNF pro-peptide variant alters its structure, influencing the interaction with SorCS2 and its biological activity, the results so far suggest the interesting idea that the BDNF pro-peptide and its naturally occurring polymorphism are negative regulators of neuronal structure and functional plasticity. A recent study provided exciting evidence for a role in vivo of the Met66 BDNF pro-peptide variant by showing that its administration to the ventral hippocampus triggers the disassembly of mushroom spines and the loss of synapses in CA1 pyramidal neurons by mobilizing different actin regulators through its interaction with the SorCS2/p75^NTR^ receptor complex, thereby disrupting cued fear extinction (Giza et al. 2018).

### TrkB signaling at dendritic spines in the absence of neurotrophins

BDNF is considered to be the prototypical neurotrophin ligand for the TrkB receptor, inducing its dimerization and activation by tyrosine phosphorylation. However, TrkB can autophosphorylate and activate its downstream signaling without BDNF in a process of transactivation depending on the Src family of tyrosine kinases (Lee and Chao 2001; Rajagopal and Chao 2006; Rajagopal et al. 2004). Transactivation of TrkB is mediated by G protein-coupled adenosine 2A or dopamine D1 receptors (Iwakura et al. 2008; Lee and Chao 2001; Wiese et al. 2007) and by an EGF-EGF receptor-induced Src-dependent pathway (Puehringer et al. 2013). In hippocampal neurons, the divalent cation zinc transactivates TrkB in a BDNF-independent manner and Src family in a kinase-dependent manner resulting in the potentiation of the mossy fiber-CA3 synapses (Huang et al. 2008). A recent study provided evidence for a role of zinc-mediated TrkB transactivation in regulating both dendritic morphology and spine density in mature primary hippocampal neurons (Zagrebelsky et al. 2018). Moreover, cocaine treatment has been shown to increase dendritic spine density in hippocampal neurons by promoting TrkB transactivation via the Sigma-1 receptor (Ka et al. 2016). Transactivation of TrkB results in biologically relevant consequences both in the healthy brain, where it regulates neuronal migration, architecture. and plasticity as well as under pathological conditions, and it should be further explored as a signaling route utilized for therapeutic approaches in neurodegenerative diseases as well as depression.

### Specific TrkB agonists

Inhibiting BDNF/TrkB signaling negatively influences dendritic spine number (Ji et al. 2010; Kellner et al. 2014; Tyler and Pozzo-Miller 2001), changes spine morphology towards a more immature phenotype (Kellner et al. 2014), inhibits long-term potentiation (Korte et al. 1995; Korte et al. 1998), and impairs learning and memory processes (Blank et al. 2016; Heldt et al. 2007). Impaired BDNF/TrkB signaling is associated with several neurological disorders, including neurodegenerative, neurodevelopmental, and neuropsychiatric diseases (for review see Duman et al. 2019; Gupta et al. 2013; Li and Pozzo-Miller 2014; Zuccato and Cattaneo 2009), characterized, among others, by dendritic spine alterations (for review see Bloss et al. 2011; Qiao et al. 2016; Xu et al. 2014). Thus, the possibility of applying BDNF as a therapeutic agent has been tested in numerous disease models, and the results show beneficial effects both in vitro and in vivo (de Pins et al. 2019; Jiao et al. 2016; Khalin et al. 2016; Zuccato and Cattaneo 2009). However, due to the poor pharmacological properties of BDNF, clinical trials could not reproduce in patients the therapeutic efficacy of BDNF observed in animal models (Lu et al. 2013). Among the possible approaches to circumvent the poor drug-like properties of BDNF, of relevance, is the development of highly selective TrkB agonists.

Small-molecule mimetics of BDNF reported to act specifically on TrkB showed beneficial effects in rescuing the symptoms of different diseases in animal models; however, only few studies investigated their ability to prevent or rescue dendritic spine pathology. Below we highlight these studies.

The most studied TrkB agonist is the 7,8-dihydroxyflavone (7,8-DHF), a member of the flavonoids family shown to cross the blood-brain barrier and to bind to TrkB with high affinity (Jang et al. 2010). In vivo studies indicate that peripheral administration of 7,8-DHF enhances emotional learning and rescues memory impairment in several rodent models (Andero et al. 2012; Choi et al. 2012; Liu et al. 2010). Specifically regarding dendritic spines, in aged rats, administration of 7,8-dihydroxyflavone improved cognitive impairment in the Morris water maze and rescued spine density in the hippocampus (Zeng et al. 2012b). Moreover, treatment with 7,8-DHF restored dendritic spine density in several brain regions associated with fear memory, including the amygdala and prefrontal cortex, and improved the performance in a fear conditioning tasks to a level similar to the one of young animals (Zeng et al. 2012a). In two different Alzheimer mouse models, dendritic spine loss could be prevented and spatial memory improved by the administration of 7,8-DHF and of its prodrug R13 (Chen et al. 2018; Gao et al. 2016). Moreover, R13 rescued dendritic spine density and promoted spine maturation in neurons of the perirhinal cortex in a mouse model of the X-linked cyclin-dependent kinase-like 5 deficiency disorders (Ren et al. 2019). These structural improvements were accompanied by the rescue of LTP and visual recognition memory (Ren et al. 2019). Finally, postnatal injection with 7,8-DHF in a mouse model of Down syndrome rescued dendritic spine number and levels of the presynaptic protein synaptophysin (Stagni et al. 2017) and was able to ameliorate the TrkB/p75^NTR^ imbalance, seen in Huntington’s disease (Garcia-Diaz Barriga et al. 2017). A second well-studied TrkB agonist, LM22A-4 (Massa et al. 2010), is a small molecule identified in silico for its high affinity specific binding to TrkB and has been shown to prevent spine loss in striatal MSNs and to improve motor deficits, in a mouse model of Huntington’s disease (Simmons et al. 2013).

In summary, the TrkB agonists have shown their propensity to rescue dendritic spine density in aging and in several disease murine models, contributing to restoring some of the typical symptoms. In contrast to methods trying to increase BDNF levels, TrkB agonists have the advantage to avert possible pleiotropic effects due to binding of BDNF to the p75^NTR^, avoiding to activate its negative modulation of spine structure and plasticity. However, it has to be mentioned that two independent studies were unable to detect activation of TrkB signaling by these compounds in vitro (Boltaev et al. 2017; Todd et al. 2014) contradicting previous reports that propose small molecules as specific TrkB agonists and suggesting that further research is required to identify and screen molecules. Interestingly, new TrkB agonist monoclonal antibodies have been identified that induce receptor activation in a manner consistent with the activation profile of BDNF (Merkouris et al. 2018; Todd et al. 2014) opening new interesting windows of opportunities which should be further developed.
